# Role of p38 MAP kinase in cancer stem cells and metastasis

**DOI:** 10.1038/s41388-022-02329-3

**Published:** 2022-04-30

**Authors:** Sriya Kudaravalli, Petra den Hollander, Sendurai A. Mani

**Affiliations:** 1 Department of Translational Molecular Pathology, The University of Texas MD Anderson Cancer Center, Houston, TX 77030 USA; 2 Rice University, Houston, TX 77030 USA

**Keywords:** Cancer stem cells, Metastasis

## Abstract

Therapeutic resistance and metastatic progression are responsible for the majority of cancer mortalities. In particular, the development of resistance is a significant barrier to the efficacy of cancer treatments such as chemotherapy, radiotherapy, targeted therapies, and immunotherapies. Cancer stem cells (CSCs) underlie treatment resistance and metastasis. p38 mitogen-activated protein kinase (p38 MAPK) is downstream of several CSC-specific signaling pathways, and it plays an important role in CSC development and maintenance and contributes to metastasis and chemoresistance. Therefore, the development of therapeutic approaches targeting p38 can sensitize tumors to chemotherapy and prevent metastatic progression.

## Introduction

**Fig. 1:**
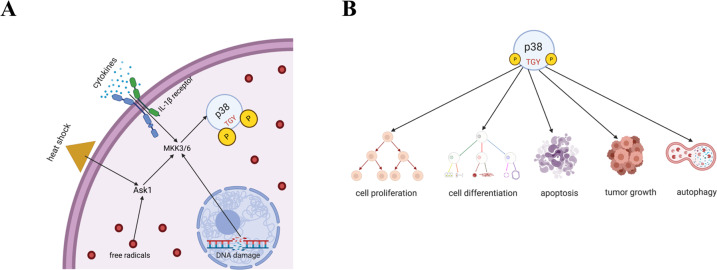
Functions of p38. **A** p38 enables cells to respond to various stimuli, including DNA damaging agents, cytokines, heat shock, and oxidative stress. **B** In addition, p38 affects cell proliferation, cell differentiation, apoptosis, autophagy, and tumor growth in ways dependent on cell type and the signaling pathways involved.

**Fig. 2:**
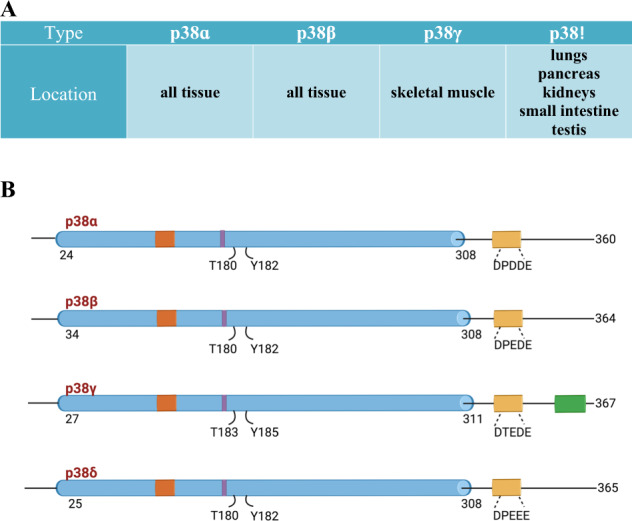
Domain structures of p38 isoforms. **A** Expression sites of different p38 isoforms. **B** p38ɑ and p38β share more sequence homology with each other than with p38γ and p38δ.

Mitogen-activated protein kinases (MAPKs) allow cells to interpret and respond to a wide variety of signals. This includes DNA damaging genotoxic agents, inflammatory cytokines, and extracellular stimuli such as changes in osmolarity, oxidative stress, and heat shock [1, 2] (Fig. 1A). The p38 MAPKs are serine/threonine-specific protein kinases characterized by a Thr-Gly-Tyr dual phosphorylation motif [3] (Fig. 1A). Four p38 isoforms have been identified (p38α, β, γ, and δ). Recently, these genes have been named as MAPK14 for p38α, MAPK11 for p38β, MAPK12 for p38γ, and MAPK13 for p38δ (Fig. 2A, B). Among them, p38ɑ and β are ubiquitously expressed and share 75% sequence identity with one another at the amino acid level. Whereas p38γ and δ have tissue-specific expression and share 70% homology with each other and 62% and 61% sequence homology with p38α, respectively [4, 5]. The expression of p38γ is limited to the skeletal muscle, whereas p38δ is expressed in the pancreas, kidneys, small intestine, testis, and lungs [4] (Fig. 2A).

All four p38 isoforms serve as nexuses for signal transduction and play crucial roles in many biological processes. This includes cell proliferation, differentiation, glucose and lipid metabolism, secretion, senescence, stress responses, apoptosis, autophagy, and cell migration (Fig. 1B) [3, 6–11]. Depending on the context, p38 proteins can be tumor-suppressive or tumor-promoting [3, 12]. The expression levels of the four different isoforms of p38 vary across different cancer types. For example, primary tumors of breast cancer, lung adenocarcinoma, and glioblastoma multiforme have similar or significantly lower MAPK11 (p38β) and MAPK12 (p38γ) protein expression levels compared to normal tissue (Supplementary Figs. 1 and 2). On the other hand, MAPK13 (p38δ) and MAPK14 (p38α) expression levels are significantly higher in the primary tumor in most cancers (Supplementary Figs. 3 and 4) [13]. Moreover, MAPK14 (p38α) T180/Y182 phosphorylation, which is induced by stress signals, is significantly reduced in primary tumor compared to normal tissue in 2 out of the three cancers tested (Supplementary Fig. 5). There is a similar survival probability between breast cancer patients with low and with high MAPK14 expression, and interestingly, MAPK14 phospho-T180/Y182 seems to have a protective effect (Supplementary Fig. 6) [14, 15]. p38α has many phosphorylation sites that regulate different downstream functions, thus different phosphorylated residues could have different correlation with survival.

p38ɑ and p38β cooperate in heart development, mitotic entry, regulatory T cell induction, and sex determination [16–19]. Similarly, p38γ and p38δ coordinate and regulate tissue regeneration and immune responses [20]. Besides, recent studies have demonstrated that p38 MAPKs regulate the behavior and function of cancer stem cells (CSCs).

CSCs are a subpopulation of tumor cells capable of self-renewal and differentiation that drive tumor initiation, recurrence, progression, and metastasis. CSCs can survive in circulation and have the potential to establish a metastatic tumor at a distant site. CSCs also contribute to the development of chemoresistance, which is responsible for 90% of treatment failures [21]. Various mechanisms contribute to the development of resistance to chemotherapy by CSCs. These cells can express drug-resistance genes such as MDR1, which encodes an energy-dependent exporter, or they can overexpress drug-efflux transporters from the ATP-binding cassette (ABC) family, which can remove chemotherapeutic agents from the cell [21]. CSCs also express aldehyde dehydrogenase 1 (ALDH1), which aids in the protection against alkylating agents such as paclitaxel [22]. In this review, we discuss the mechanisms through which p38 attenuates and augments CSC properties contingent on the cancer type and how p38 can be targeted to overcome chemoresistance and block metastasis.

## Role of p38 in CSCs

### Enrichment of CSCs by p38

Anywhere between 0.1 and 25% of the total cell population in a solid tumor comprises of CSCs [23, 24]. CSCs can be identified and enriched using various cell surface markers, such as CD44^hi^/CD24^lo^ for breast cancer [25], CD133 for brain cancer [26], and many others [23, 27]. CSCs divide asymmetrically and generate one daughter cell retaining the stem-cell identity, while the other cell differentiates and is often highly proliferative [23]. Patient-to-patient, the CSC populations are highly variable. This is because CSC properties are influenced by tumor-specific genetic aberrancies, the stage of disease progression, and the types of drugs used to combat tumor growth [28].

Besides the transformation of normal stem cells to cancer stem cells, CSCs can also come from differentiated cancer cells through the activation of the epithelial-to-mesenchymal transition (EMT) [23, 29]. Several studies have shown that high levels of expression of EMT-inducing transcription factors ZEB1/2, SNAI1/2, TWIST1/2, and FOXC2 in cancer cells trigger the expression of stemness factors such as SOX2, BMI1, and OCT4 and enhance the ability to self-renew and to form mammospheres, all of which are characteristics of CSCs [29–32]. In pancreatic cancer cells, mechanical stress upregulates EMT transcription factors, activates p38, and enhances cell migration [33]. Although the transcription factors that promote EMT and enrichment of CSCs are known, these proteins are hard to target pharmacologically relative to kinases like p38.

**Fig. 3:**
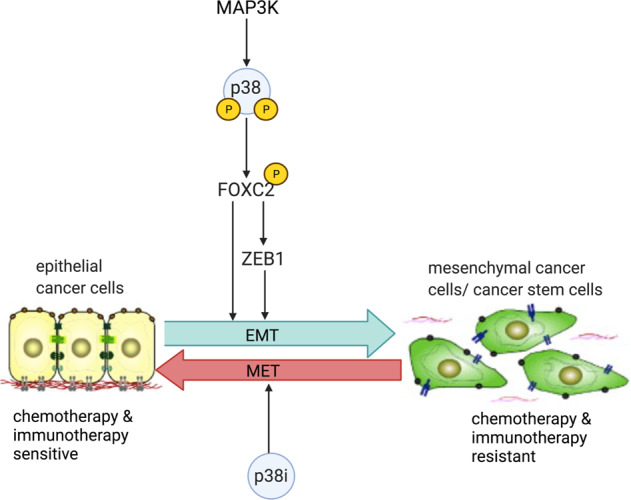
Schematic of the p38-FOXC2 signaling axis. The p38-FOXC2 signaling axis is critical in the formation of CSCs. p38, regulated by many upstream kinases, phosphorylates FOXC2, which then activates ZEB1. Activated FOXC2 and ZEB1 promote the EMT, initiating metastasis and conferring stemness in cancer cells. p38 inhibition reverses the EMT and inhibits stemness in cancer cells.

In human mammary epithelial cells induced to undergo EMT, p38α phosphorylates FOXC2 at serine 367, stabilizes FOXC2, confer stem-cell attributes in vitro, and metastatic competence in vivo [12, 34] (Fig. 3). Additionally, inhibition of p38α blocks stem-cell properties such as sphere-forming potential and the CD44^hi^/CD24^lo^ stem-cell marker profile (Fig. 3). Besides, depletion of p38α from epithelial cells by shRNA knockdown blocks these cells’ ability to undergo EMT in response to Snail or Twist [34], suggesting that many EMT- and CSC-promoting signals are potentially transmitted through p38.

Overexpression of p38γ in the luminal A breast cancer cell line MCF7 increases CSCs and tumorspheres [35, 36]. Similarly, knockdown of p38γ expression decreases the frequency of the CSC population and blocks tumorsphere formation of breast cancer cell lines MCF7-ErbB2 and BT47, which express HER2 oncogene [36]. In the same way, knockdown of p38γ from triple negative breast cancer (TNBC) cell lines MDA-MB 231 and MDA-MB 468 significantly reduces sphere formation, suggesting that p38γ is also capable of manipulating the CSC population [37]. In addition, silencing of p38γ significantly decreases the key CSC drivers Nanog, OCT3/4, SOX2, and CD44 [37]. Interestingly, p38γ expression alone is sufficient to induce expansion of the CSC population, likely due to its direct stimulation of Nanog expression via c‐Jun‐mediated binding to the activator protein 1 (AP1) site of the *Nanog* promoter [37]. Nanog then transcriptionally induces the expression of *SOX2* and *OCT3/4*, leading to CSC expansion and inducing TNBC progression [37]. Thus, p38 acts to manipulate CSC properties in multiple breast cancer subtypes.

In addition to serving as an upstream regulator of CSC-inducing transcription factors, p38 also functions as a downstream target of transcription factors through different signaling axes. For example, in response to chemotherapy, the hypoxia-inducible factor HIF1 induces increases in DUSP9 and decreases in DUSP16 expression, leading to activation of the p38 signaling pathway in TNBCs [38]. Activation of p38 then increases the expression of Nanog and KLF4 through phosphorylation and inactivation of ZFP36L1, thus increasing the CSC pool [38]. This complex dual involvement of p38 is critical in regulating CSC maintenance and expansion in breast cancer.

In the head and neck squamous cell carcinoma cell line SCC-131, inhibition of p38 significantly reduces tumor spheroid formation and decreases the expression of the CSC markers SOX2, OCT4, KLF4, c-MYC, and CD44, indicating that p38 plays a key role in the maintenance of stemness properties in this cancer type [39]. Activation of the p38 pathway also enhances the survival of colorectal CSCs under hypoxia and serum-depletion conditions, which are two well-defined capabilities of CSCs [40]. The interaction of neuropilin 1 (NRP-1) and VEGF-A initiates a cascade involving GIPC1, SYX, RhoA/ROCK, and MEK3/6 to activate p38 necessary to enrich epidermal CSCs, as p38 depletion reduces CSC spheroid formation and invasion [41].

KLF family of zinc finger transcription factors KLF4 activates the p38 signaling pathway in osteosarcoma, promoting cancer stemness [42]. Not only does KLF4 overexpression cause a substantial increase in osteosphere formation and dimension, but it also significantly increases the transcription of stem-cell-associated genes, including *CD133*, *ALDH1A1*, and *ABCG2* [42]. Interestingly, when the expression of p38 is inhibited using siRNA, the KLF4 induced spheres are also reduced, suggesting that the p38 signaling pathway activates the expression of stem-cell transcription factors and downstream transducers of these transcription factors [42]. Downregulation of p38γ using shRNA in breast cancer cells overexpressing ErbB2 decrease the alcohol-induced increase in CSCs, mammosphere formation, and migration and invasion [43]. The cytokine IL-17 activates the p38 signaling pathway, thereby stimulating self-renewal of ovarian CD133^+^ CSCs, and IL-17-promoted self-renewal is compromised upon p38 inhibition [44]. In hepatocellular carcinoma, long-term tobacco exposure increases IL33 expression in liver tissues. The increased IL33 promotes p38 activation, inducing EMT and increasing the levels of CSC markers CD133, Nanog, and OCT4, and this can be abrogated by p38 inhibition [45].

### Role of p38 in tumor-initiating vs. metastatic CSCs

Tumor-initiating CSCs (tiCSCs) and metastatic CSCs (mCSCs) are the two predominant pools of CSCs within tumors. While tiCSCs are responsible for the development of heterogeneous lineages of cancer cells that comprise the primary tumor [46], the mCSCs possess migratory and invasive capabilities, survival capacities in circulation, and metastatic potential [29]. Although mCSCs and tiCSCs share some phenotypic similarities, mCSCs are shown to accumulate new genetic alterations at secondary sites that make them resistant to chemotherapies that are effective against tumor-initiating CSCs [47]. Stemness plays an important role in metastasis, specifically in relation to the expression of various integrins and the formation of circulating tumor cells.

In breast cancer, the expression of integrin αvβ3 induces CSC properties, including tumorsphere formation [48] and the integrin subunits β1 and β3 are used as CSC markers [49]. The β3 is necessary and sufficient for the CSC phenotype in lung, prostate, and breast cancers [48, 50, 51]. Expression of those same integrins, αvβ3, β1, and β3, contributes to lymph node and bone metastases [52–57]. Several studies reported that most circulating tumor cells have CSC-like features [58–62]. Because p38 promotes CSC properties and stemness is implicated in metastasis, p38 might specifically enrich mCSCs as opposed to tiCSCs. Moreover, mCSCs are characterized by CXCR4 expression, which promotes metastasis by activating p38, thus suggesting the involvement of p38 in the production of mCSCs in particular [63].

Activation of the EMT program also induces stemness and p38 signaling [34]. By concomitantly activating the EMT and p38 signaling, cancer cells gain a migratory and mesenchymal phenotype, which is required for metastasis [64, 65]. Inhibition of p38 inhibits FOXC2 and reverts prostate cancer cells from a mesenchymal phenotype with metastatic properties to an epithelial phenotype that is incapable of developing metastatic growth [66]. In addition, p38 inhibition impedes metastasis in breast cancer but not primary tumor growth [34]. The former is mediated by mCSCs, whereas the latter is underpinned by tiCSCs. These findings are strong indicators that p38 proteins play a critical role specifically in mCSCs.

### p38 diminishes the CSC population

Although substantial evidence supports a pro-CSC role for p38, some findings argue that p38 can attenuate CSC properties and decrease the CSC population [67–73]. This warrants further characterization of the contributions of each of the four p38 isoforms to CSC properties. The p38/nuclear factor (NF)-kB/Snail signaling pathway is involved in caffeic acid-induced inhibition of CSC properties and migratory capacity of malignant human keratinocyte HaCaT cells [67]. In caffeic acid-treated HaCaT cells, phosphorylation of p38 is increased and NF-kB binding to the *Snail* promoter is decreased, resulting in the downregulation of Snail, a transcription factor linked to the acquisition of CSC-like characteristics [67]. Caffeic acid-treated HaCaT cells also have attenuated sphere-forming capacity and decreased expression of *CD34* and the keratin encoding *K5*, which are markers of CSCs and skin stem cells, respectively [67]. Thus, through this pathway, p38 acts as an upstream regulator of NF-kB and Snail to reduce CSC properties in skin cancer.

A non-anticoagulant heparan sulfate hexasaccharide sequence, HS06, selectively inhibits CSC self-renewal and induces apoptosis in breast, colorectal, and pancreatic CSCs [68]. HS06 inhibition of CSCs is dependent on early and sustained activation of p38α and β, which inhibits TCF4-mediated signaling and, therefore, CSC self-renewal [68]. When p38 is inhibited using SB203580 in cells treated with HS06, sphere formation is enhanced, and CSC markers (CD44 and CD133) and self-renewal factors (c-MYC and BMI1) are expressed at higher levels than in cells treated with HS06 alone [68]. Glycosaminoglycans (GAGs) are also essential regulators of stemness. Like HS06, G2.2, a sulfated non-saccharide GAG mimetic, induces early and sustained activation of p38 in human colorectal HT29 spheroids [69]. Importantly, pharmacological inhibition of p38 with SB203580 reverses G2.2-mediated inhibition of CSC self-renewal, as evidenced by the increased 3D spheroid formation and expression of CSC and self-renewal markers [69]. These results imply that p38α and β can also inhibit CSC self-renewal under certain circumstances.

Activation of the p38γ and δ isoforms abolishes the CSC properties and tumor-initiating ability of non-small cell lung cancer (NSCLC) cells through ubiquitination and degradation of stemness proteins SOX2, OCT4, Nanog, KLF4, and c-MYC through MK2-mediated phosphorylation of Hsp27, a fundamental component of the proteasomal degradation machinery [70]. The inactivation of p38 induces the upregulation of stemness proteins in NSCLC cells, causing them to acquire CSC properties [70]. WIP1, a p38 phosphatase frequently overexpressed in cancer, promotes stemness-related protein expression and CSC properties by inhibiting p38 activity in NSCLC cells [71].

p38 also regulates ubiquitin-mediated degradation of stemness factors in glioma CSCs, as activation of the p38 pathway leads to reduced epidermal growth factor receptor (EGFR) surface expression via ubiquitin ligase-mediated degradation of EGFR, thereby attenuating the sphere-forming and self-renewal capacities of the CSCs [72]. Upon p38 inhibition, the number of undifferentiated CSCs increases [72]. In contrast to the reported enhancement of CSC properties upon overexpression of p38γ in MCF7 cells [36], in another CSC model resulting from ectopic overexpression of the stem-cell marker Nanog in MCF7 cells, activation of p38 and AMPKα pathways result in inhibition of CSC growth and induction of apoptosis [73]. In addition, in a breast cancer model, activation of p38 from extracellular matrix-dependent compressive forces promoted a differentiated cell phenotype [74]. Although there is limited evidence for a CSC-suppressive role of p38, further study of the involvement of the different p38 isoforms in CSC suppression is warranted.

## Mechanisms of p38-mediated chemoresistance

Metastasis is the primary cause of cancer-related mortality, accounting for more than 90% of cancer-related deaths [75]. Although chemotherapy remains the main treatment for many types of cancer, chemoresistance is almost always observed in patients with metastatic cancer. During treatment, some tumor cells transform into CSCs, become chemoresistant, and acquire the ability to become metastatic. Not only do p38 proteins regulate signaling that contributes to CSC maintenance and expansion, but these proteins are also directly linked with the mechanisms that result in chemoresistance.

### Drug efflux

**Fig. 4:**
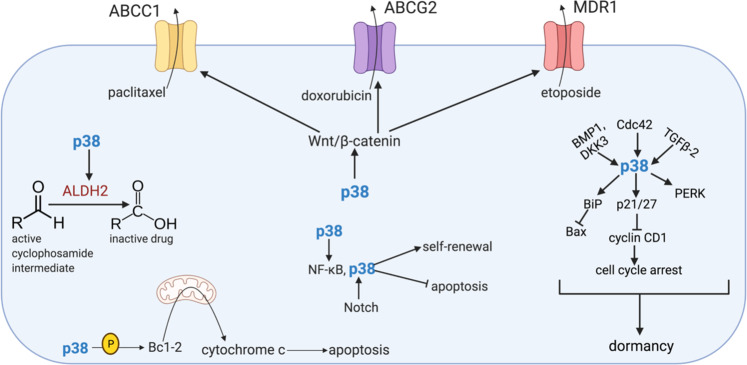
Mechanisms of chemoresistance in cancer stem cells. Upon p38 activation, CSCs are maintained in a non-proliferating, quiescent state for long periods, thus escaping chemotherapies that target rapidly dividing cells. p38 regulates the expression of ALDHs, such as ALDH2, which detoxify the metabolites of chemotherapeutic agents, rendering them ineffective. The WNT/β-catenin signaling pathway, promoted by p38, mediates chemoresistance via the upregulation of ABC transporter pumps such as ABCC1, ABCG2, and MDR1, which remove chemotherapy agents from inside the cell. These ABC transporters are also directly upregulated by p38. Moreover, the Notch and NF-κB signaling axes promote self-renewal and inhibit apoptosis of CSCs even in the presence of chemotherapy drugs. The activity of NF-κB is stimulated by p38, and Notch activates p38. Additionally, p38 phosphorylates BCL-2, promoting apoptosis.

ABC transport proteins ABCB1, ABCC1, ABCG2, and MDR1 are expressed within CSCs [76] (Fig. 4). ABC transporters shield cells from harmful toxins and xenobiotics [77]. For example, MDR1 prevents the entry of foreign toxins into the growing fetus and sensitive organs such as the kidneys and brain, and ABCG2 blocks toxins from invading the mammary gland, hematopoietic stem cells, and the blood-brain barrier [77]. However, CSCs use these same ABC transporters to pump chemotherapy agents (e.g., paclitaxel, doxorubicin, vinblastine, etoposide, and colchicine) out of the cells [77]. Inhibition of p38 by BIRB796 can reverse multidrug resistance induced by ABCB1, ABCC1, and ABCG2 by directly inhibiting their ATPase and transport functions [78] (Fig. 4). Moreover, inhibition of p38 by SB202190 reduces MDR1 expression levels in human gastric cancer cells and sensitizes them to the chemotherapeutic agent vincristine, suggesting a role for p38 in drug-efflux-mechanism-driven chemoresistance [79] (Fig. 4).

### ALDH activity

Cytosolic ALDHs oxidize intracellular aldehydes and convert them into carboxylic acids [80] (Fig. 4). CSCs express high levels of ALDHs, which confer chemoresistance to CSCs by detoxifying the aldehyde intermediates produced in CSCs treated with certain chemotherapy agents and protecting CSCs from the noxious effects of elevated reactive oxygen species levels, which result from oxidative stress generated by chemotherapy drugs [81]. Resistance to paclitaxel and epirubicin is seen in breast tumor samples enriched in CSCs with high levels of ALDH activity [82]. Additionally, when compared to counterparts that do not express these dehydrogenases, ALDH-positive cells from lung cancer cell lines have higher resistance to multiple chemotherapy agents [83]. Detoxification of other chemotherapeutics, such as the alkylating agent cyclophosphamide, also occurs through ALDH activity. Once inside the cell, the pro-drug form of cyclophosphamide is converted to 4-hydroxy cyclophosphamide and then to aldophosphamide [76]. As shown by studies in leukemia cells, aldophosphamide is detoxified by ALDHs specifically in the CSC subpopulation, making this enzyme a primary driver for CSC chemoresistance [76] (Fig. 4). In acute myeloid leukemia, TGF-β1-mediated p38 activation induces ALDH2 expression and chemoresistance [84] (Fig. 4). It is likely that p38 regulates the expression of ALDHs in other cancers, suggesting that p38 inhibition could reduce the expression of ALDHs and sensitize CSCs to chemotherapy.

### Quiescence and dormancy of CSCs

Quiescence by cancer cells is reversible, and a quiescent cell may reenter the cell cycle in response to physiological cell stimuli [85]. Dormant tumors consist of CSCs in a non-proliferating state, and these quiescent tumor cells may persist for long periods at metastatic sites [85]. Activation of p38 signaling is dependent on the secretion of BMP7 and promotes dormancy and quiescence in prostate CSC-like cells [86] (Fig. 4). Bone-secreted factors DKK3, vasorin, and neogenin induce dormancy in prostate cancer cells via p38 activation as well [87] (Fig. 4). p38 activation also induces dormancy in squamous carcinoma cells [88]. p38 signaling allows these dormant tumor cells to resist chemotherapy by activation of pro-survival mechanisms driven by upregulation of the protein kinases PERK and BiP, which prevent the activation of Bax, a pro-apoptotic protein [88] (Fig. 4). In addition, whereas a high ratio of ERK to p38 induces tumor growth, a low ratio results in tumor growth arrest [89]. In a head and neck squamous carcinoma cell model, TGF-β2 signaling in the bone marrow activates p38α and β, resulting in a low ERK to p38 signaling ratio and dormancy of malignant disseminated tumor cells [90] (Fig. 4). Activation of p38 can also act through other pathways. For example, high levels of p38 result in CDC42 expression, which induces p21/p27 and represses cyclin D1 expression, thereby causing cell-cycle arrest [11, 91, 92] (Fig. 4).

Because chemotherapeutics target rapidly dividing cells, the slow-cycling, quiescent phenotype of CSCs renders them resistant to conventional therapy. For example, slow-cycling glioma stem cells in a transgenic mouse model survive the alkylating drug temozolomide and eventually cause recurrence [93]. However, when the slow-cycling CSCs are removed from the tumor, the tumor becomes chemosensitive, and the mice survive for a much longer time [93]. Thus, it is possible that p38 inhibition could push CSCs out of their quiescent state, making them more susceptible to chemotherapy-induced death.

### Defective DNA repair

Mechanisms such as proofreading, mismatch repair, nucleotide excision repair, and base excision repair fix errors introduced during DNA replication. Because cancer cells rapidly divide, they are often in S phase, which is a vulnerable phase for DNA damage induced by chemotherapeutic agents (including the common analogs of cisplatin, carboplatin, and oxaliplatin) [76]. Due to defective DNA repair pathways, most cancer cells cannot recover from the DNA damage-induced stress and undergo apoptosis. In contrast, CSCs have increased levels of checkpoint kinases and greater DNA repair capacity than a typical cancer cell; consequently, CSCs avoid death usually induced by chemotherapy [94]. Activation of p38ɑ, which is seen in many CSC populations, leads to the activation of the G2 checkpoint in tumors treated with agents such as temozolomide [95]. Activation of the G2 checkpoint increases the fidelity of DNA repair and thus the maintenance of CSCs. p38ɑ also maintains genomic stability and enables CSC survival through activating the ATR-Chk1 signaling axis, promoting DNA replication and repair [96]. Additionally, p38ɑ directly phosphorylates and activates CtIP, which is responsible for DNA double-strand break resection and proper DNA repair [96]. Moreover, activated p38 MAPK signaling increases the expression levels of BRD4 and 53BP1 (key DNA damage response factors that promote nonhomologous end joining repair), thereby repairing damaged DNA and protecting cells from apoptosis [97]. p38 inhibition could be utilized to prevent the activation of cell-cycle checkpoints, particularly the G2 checkpoint, and DNA repair pathways in order to increase the accumulation of DNA damage in CSCs, resulting in induction of apoptosis.

### Signaling gone awry

Many signaling pathways involving p38 lead to chemoresistance. The WNT/β-catenin signaling pathway, which is promoted by p38β via phosphorylation of LRP6, is required for normal stem and CSC self-renewal in numerous cell types. This signaling has also been shown to contribute to chemoresistance [77, 98] (Fig. 4). The Notch signaling pathway, which plays an important role in tumor progression and metastasis, is also implicated in CSC maintenance and chemoresistance [99]. Notch proteins, specifically Notch1, stimulate the p38 pathway, which in turn results in enrichment of the CD133^+^ CSC population [100] (Fig. 4). Moreover, NF-κB, a key regulator of the inflammatory response with activity that is enhanced by p38, contributes to chemoresistance [101] (Fig. 4). On the other hand, phosphorylation of BCL-2 by p38 diminishes the anti-apoptotic potential of BCL-2, thus making CSCs more susceptible to death from chemotherapy [102] (Fig. 4).

p38 plays various roles in other forms of therapy resistance like in immunotherapy. On one hand, in breast cancer clinical samples and mouse models, GS-CSF signaling through p38 activates myeloid cell ARG1 expression, inhibiting antitumor immunity from T cells [103]. This forms an immunosuppressive tumor microenvironment which renders immunotherapy ineffective. On the other hand, some studies show that p38 does not contribute to immunotherapy resistance. For example, a study found that through the HMGCR/p38 signaling pathway, AMPK suppresses tumor progression by downregulating PD-1 in regulatory T cells [104].

In summary, p38 is involved in many chemoresistance mechanisms and pathways. Because p38 activation leads to CSC enrichment and CSCs are linked to chemoresistance, p38 inhibition could be used to overcome chemoresistance. p38 inhibition not only decreases expression of stemness factors and compromises intravasation, distant colonization, and survival of circulating tumor cells, but it would also push metastatic, mesenchymal-like tumor cells into an epithelial state by inducing the mesenchymal-to-epithelial transition, thereby rendering them chemosensitive [34, 105, 106].

## p38 combination treatments to overcome chemoresistance

**Fig. 5:**
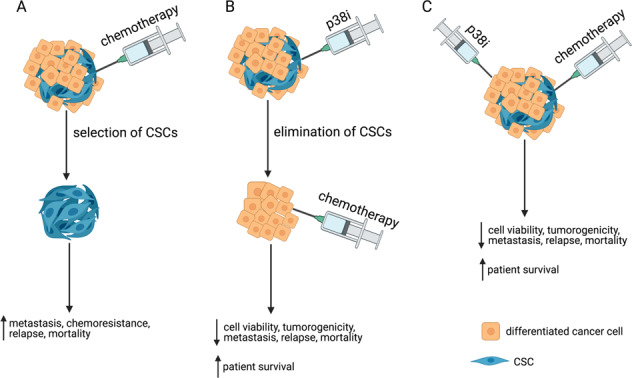
p38 inhibition sensitizes tumor cells to standard chemotherapy. Schematic of the effects of **A** chemotherapy alone, **B** pre-treatment with the p38 inhibitor SB203580 followed by chemotherapy, and **C** co-treatment with the p38 inhibitor SB203580 and chemotherapy. The combination treatments overcome the chemoresistance of tumor cells by inhibiting the formation of CSCs and decreasing cell viability and tumorigenicity. This can lead to lower rates of cancer recurrence and higher survival rates.

The efficacy of chemotherapy drugs depends on the cancer type and on the stage of cancer. Therefore, it is important to identify the chemotherapy drugs that have increased efficacy in combination with p38 inhibition. In neuroblastoma cells, treatment with etoposide alone activated the p38 pathway, thus increasing the number of neurospheres, which are the CSCs of neuronal origin, and upregulating MDR1, which directly contributes to chemoresistance [2, 79, 107] (Fig. 5). However, pre-treatment and co-treatment with the p38 inhibitor SB203580 dramatically sensitizes neuroblastoma cells to etoposide, strongly reducing the dosage needed to inhibit tumorigenicity and neurosphere formation [107] (Fig. 5). p38 inhibition increases the sensitivity of lymphoma cells to etoposide as well [108].

Combination of the p38 inhibitor LY479754 and temozolomide heightens the vulnerability of glioma cells to chemotherapy [109]. p38 inhibition also increases sensitivity to cisplatin chemotherapy in head and neck squamous cell carcinoma cells [110]. In gastric cancer cells, the combination of SB203580 with doxorubicin significantly reduces cell viability and increases cell death, as inactivation of the p38 signaling pathway results in an increase in expression of Bax, an apoptotic protein, and a concomitant decrease in the expression of BCL-2, an anti-apoptotic protein [111]. Likewise, p38 inhibition significantly increases colorectal cancer cell sensitivity to 5-FU. Similarly, combination of SB203580 with 5-FU significantly reduces cell viability and increases cell death and cellular caspase activity compared to 5-FU treatment alone and the SB203580 sensitizes cancer cells to 5-FU through an increase in Bax expression [112]. p38 inhibition also increases the sensitivity of colorectal cancer cells to irinotecan [113], which alone results in activation of p38, as demonstrated by increased levels of phosphorylated p38 [113]. p38 activation enhances CSC properties and DNA repair and inhibits autophagy and cell death, hence conferring chemoresistance [113, 114].

A p38 inhibitor/chemotherapy combination also has potential in gynecological cancers. In TNBC lines, the combinations of p38 inhibitors LY2228820, VX-702, or PH-797804 with gemcitabine and with epirubicin reduce TNBC cell proliferation to a greater extent than either of the two chemotherapy drugs individually [115]. This chemosensitization may be due to a loss of CSC features, as demonstrated by a decrease in the levels of the stemness marker FOXC2 and an increase in the epithelial marker E-cadherin resulting from inhibition of p38 activity [115]. In a mouse model of breast cancer, inhibition of p38 cooperates with cisplatin to target tumor cells [116]. Importantly, the breast tumors were smaller and in a less advanced stage at the end of the study period in the case of the combined therapy compared to cisplatin alone [116]. Moreover, in patient-derived xenografts from TNBC and luminal breast tumors, p38ɑ inhibition enhanced the anti-tumoral effect of taxanes alone, leading to greater tumor clearance [96]. Combination of p38ɑ inhibition with taxane-based chemotherapies such as paclitaxel and docetaxel increased DNA damage, missegregation, and aneuploidy in cancer cells, leading to tumor regression and prevention of tumor relapse [96].

There is also evidence from a human clinical trial that the addition of ralimetinib, a p38 inhibitor, to gemcitabine and carboplatin results in a modest improvement in progression-free survival in ovarian cancer patients compared to administration of chemotherapy alone [117]. Furthermore, metformin combined with a p38 inhibitor improves cisplatin sensitivity in cisplatin-resistant ovarian cancer cells [118]. As recently reviewed, p38 inhibition appears to make hybrid epithelial/mesenchymal cells, which possess enhanced stem-like properties and are known to be chemoresistant, sensitive to taxanes and anthracyclines [119]. Although further investigation is needed, there is a plenty of in vitro, in vivo, and clinical trial evidence that inhibition of p38 sensitizes several different cancer types to standard-of-care chemotherapy treatment.

## Conclusions and future perspectives

In most cancers, p38 inhibition decreases the expression of stemness factors and compromises intravasation, distant colonization, and survival of circulating tumor cells. p38 inhibitors can also be employed in cancer treatment regimens to circumvent and overcome chemoresistance. Importantly, systemic p38 inhibition has very minimal side effects. Combining p38 inhibitors with standard-of-care chemotherapies will allow treatment with lower doses of these toxic drugs while eliminating residual CSCs and circulating tumor cells.

Further studies of p38 functions are critical because, in certain scenarios, p38 activation may be desired. It is important to understand in what tissues and cancers p38 activation enhances or diminishes the CSC population. The opposing roles of p38 may result from its functions in different signaling axes. As a result, targeting p38 could sometimes produce the opposite of the intended effect, thus posing a barrier to incorporating p38 inhibition as a pharmacological strategy in diseases involving stem cells and CSCs. Despite this limitation, p38 inhibitors have been successfully implemented in cancer treatment regimens to overcome chemoresistance when used in conjunction with various chemotherapeutics.

Further studies in preclinical models should explore whether pre-treatment or co-treatment with p38 inhibitors will effectively increase sensitivity to chemotherapeutic agents. Determination of the correct treatment schedule is important for the successful treatment of cancer patients. Thus, whether p38 signaling should be inhibited before additional treatments are given or if simultaneous targeting is as or more efficacious needs to be determined. We speculate that, in most cases, reprogramming the CSCs before chemotherapy will increase efficacy and prevent the enrichment of CSCs. p38 inhibition may not induce chemosensitivity in certain cancer cells, such as those with extreme mesenchymal phenotypes, which are associated with very high levels of chemoresistance [120–122]. In fact, particular chemotherapy drugs themselves, such as doxorubicin, can activate p38 in certain cell lines, potentially enhancing CSC properties and chemoresistance in turn [111]. Therefore, certain cancers might respond to initial treatment with chemotherapy followed by p38 inhibitor treatment. There is also great promise in combining p38 inhibition with targeted therapy and immunotherapy in CSC-enriched tumors. In summary, using a p38 inhibitor in combination therapy may allow dose reductions of highly toxic chemotherapy drugs and may prevent resistance to targeted and immunotherapy to eliminate CSC-enriched cancers and prevent metastasis.

## Supplementary information

Supplementary figures and legends
