# Supplementary material for: Role of p38 MAP kinase in cancer stem cells and metastasis
Source: Oncogene. 2022 Apr 30;41(23):3177–85. doi: 10.1038/s41388-022-02329-3 (PMC9166676; doi:10.1038/s41388-022-02329-3)
Supplement: Supplementary file 1 — Supplementary figures and legends [file 41388_2022_2329_MOESM1_ESM.docx]

A.


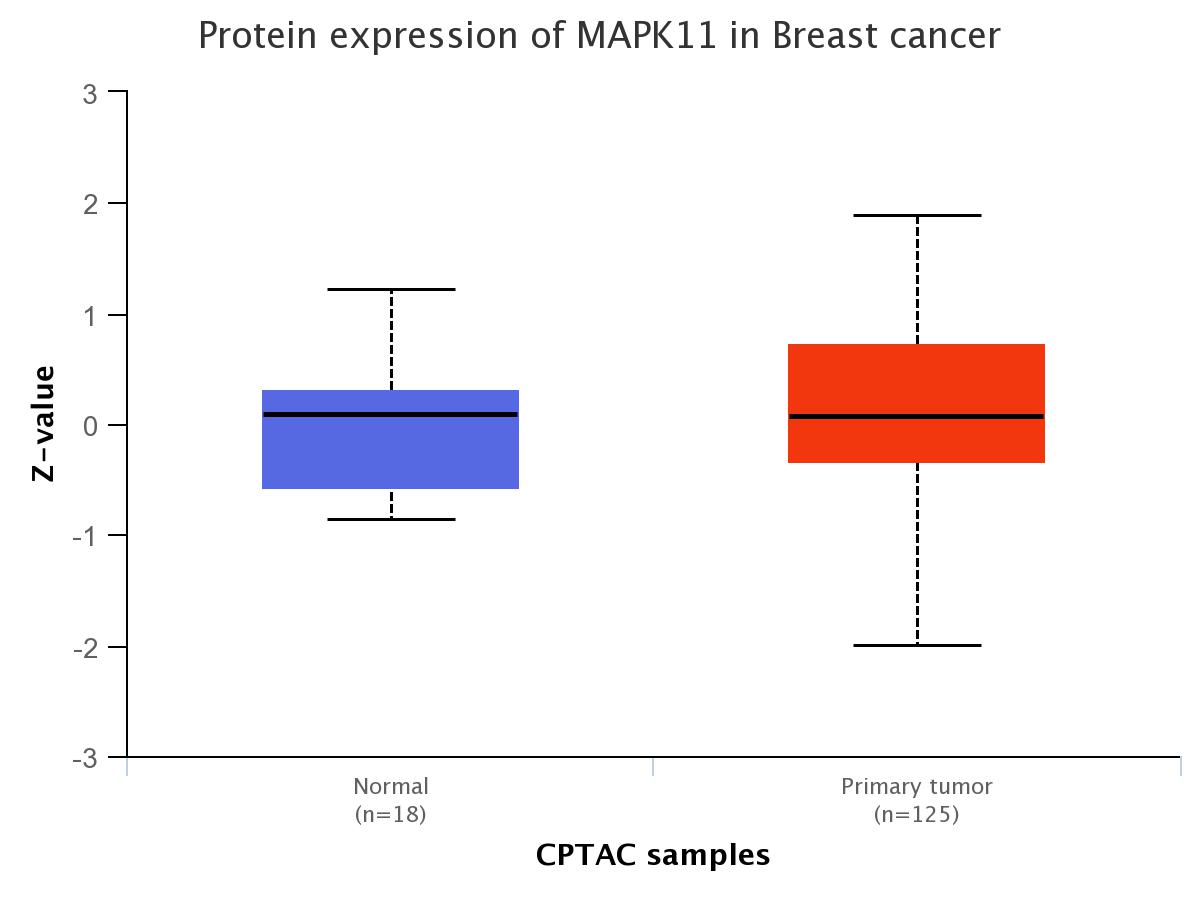


B.

p = 0.8

B.


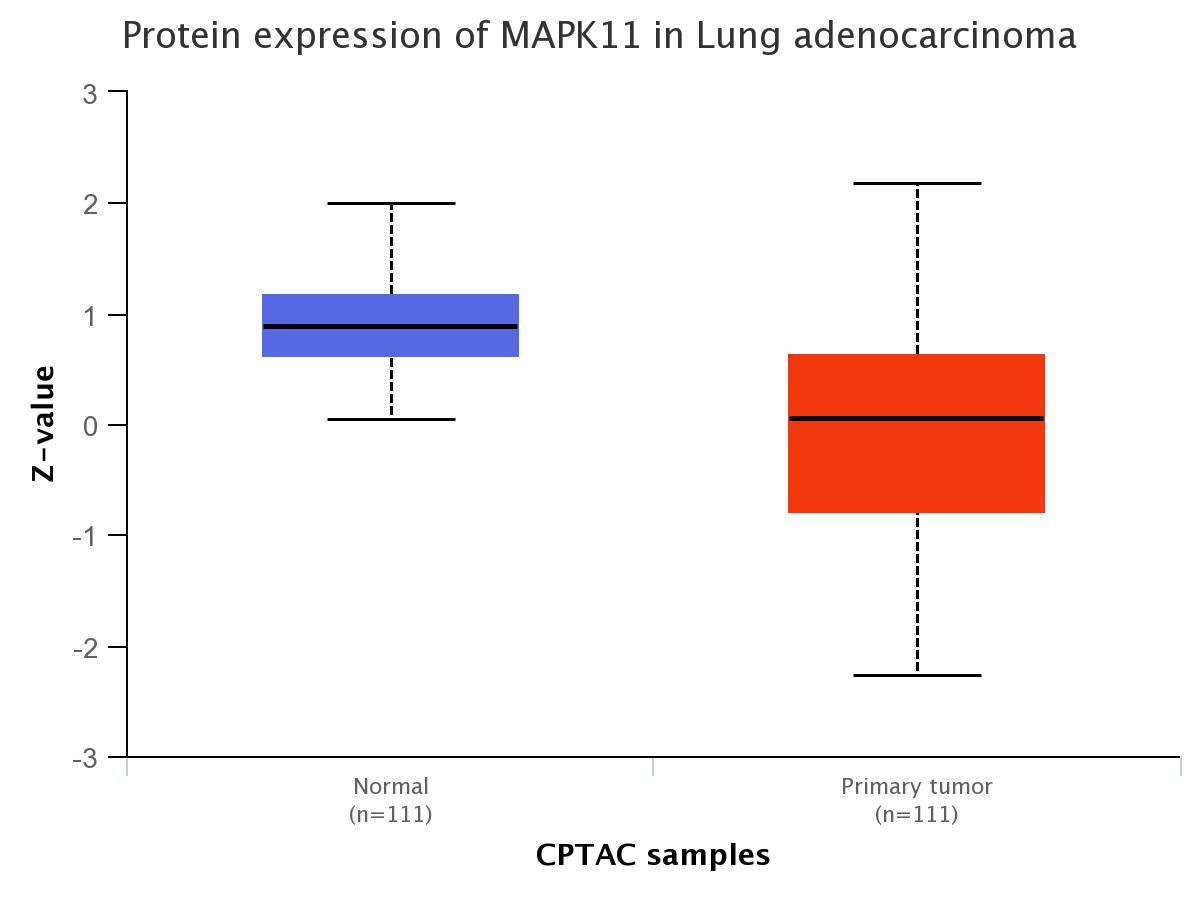


p = 2.2 x 10^-18^

C.

C.


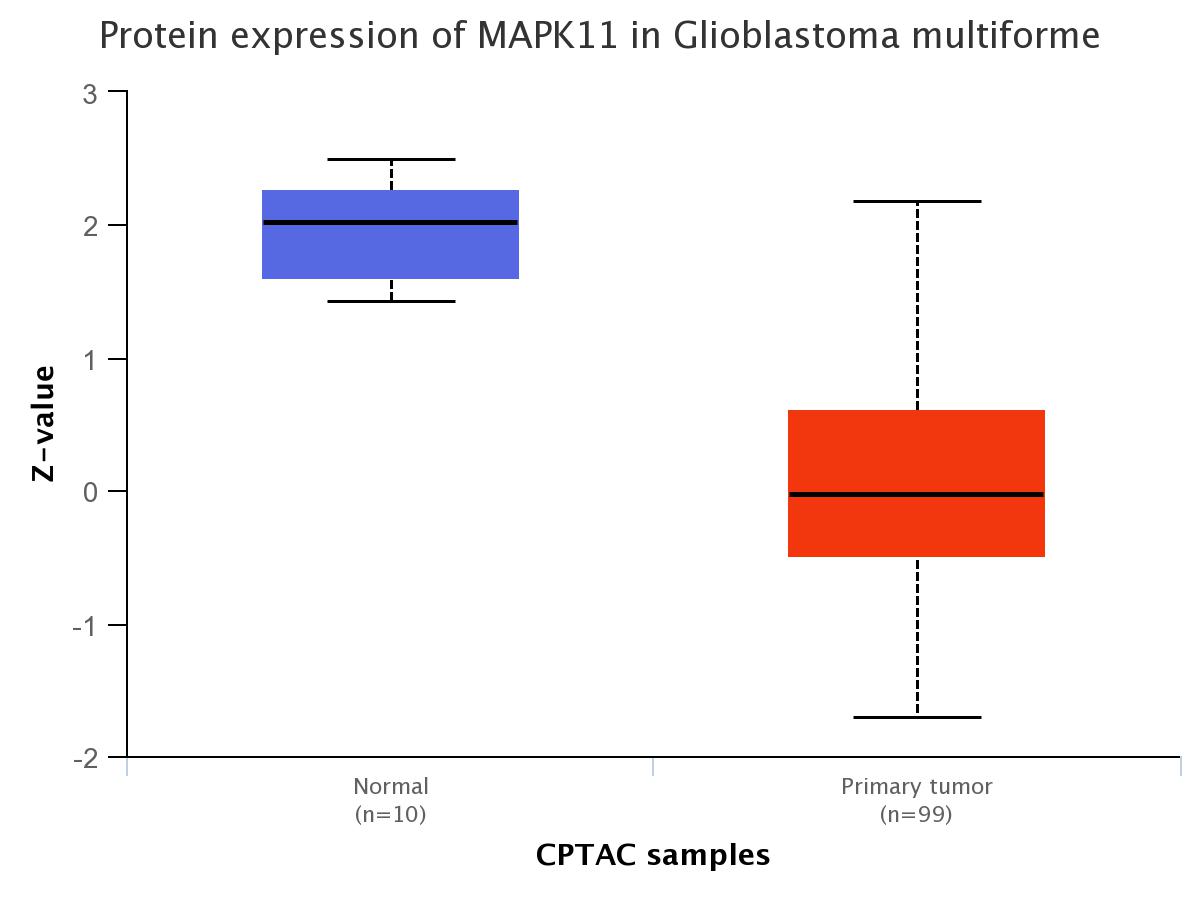


CPTAC data was derived from UALCAN.

p = 3.1 x 10^-11^

**Supplemental Figure 1:** Total protein expression levels of MAPK11 in **A.** breast cancer, **B.** lung adenocarcinoma, and **C.** glioblastoma multiforme. CPTAC data was derived from UALCAN.

A.

**
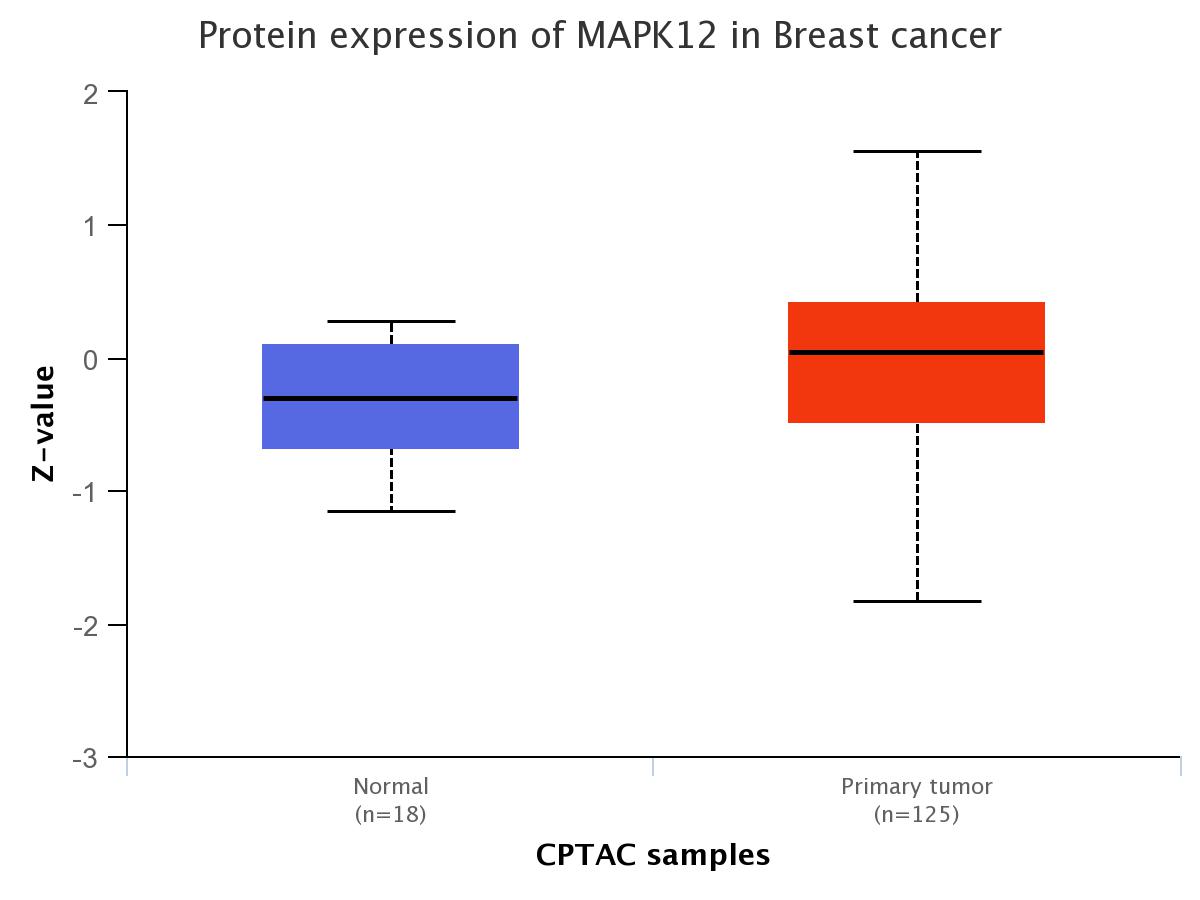
**

p = 0.3

B.

B.


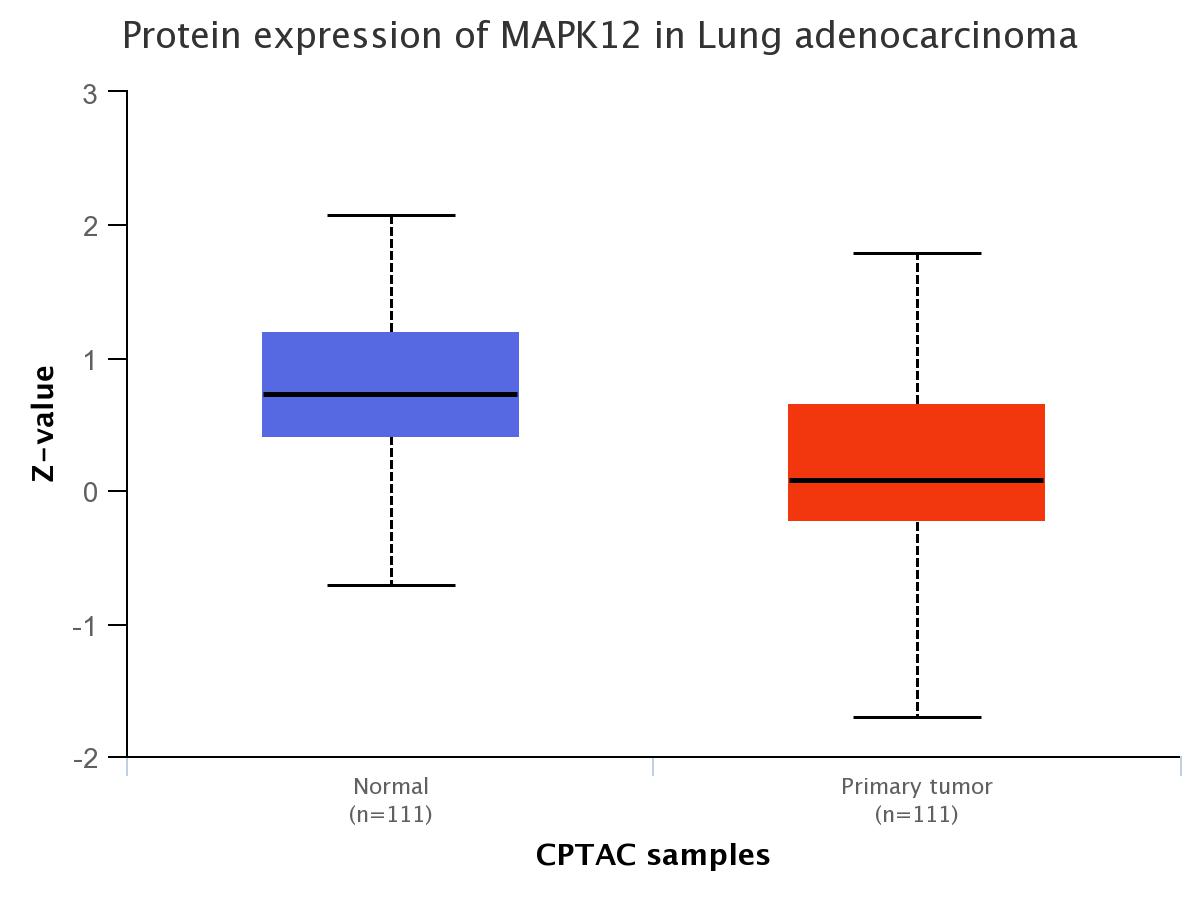


C.

p = 2.8 x 10^-12^

C.


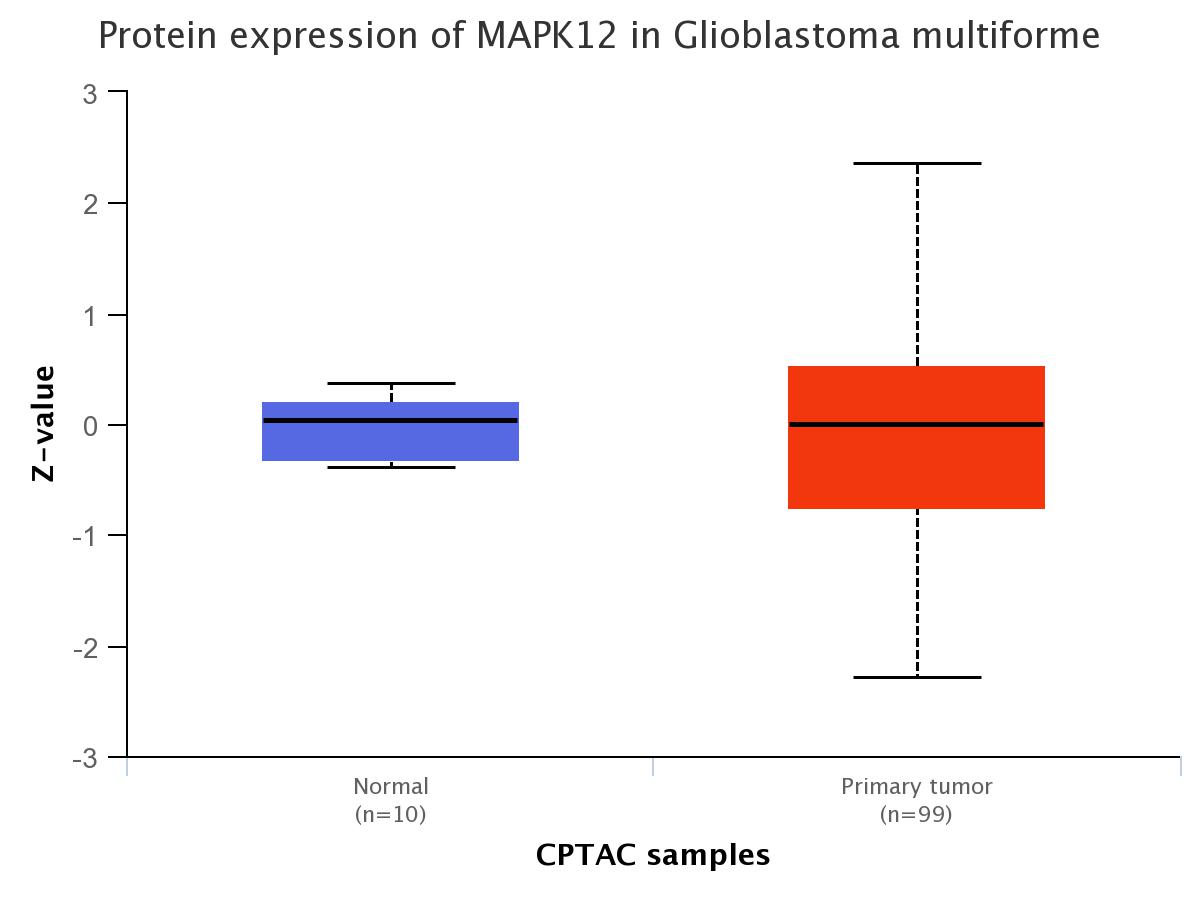


multiforme. CPTAC data was derived from UALCAN.

p = 0.3

**Supplemental Figure 2:** Total protein expression levels of MAPK12 in **A.** breast cancer, **B.** lung adenocarcinoma, and **C.** glioblastoma multiforme. CPTAC data was derived from UALCAN.

A.


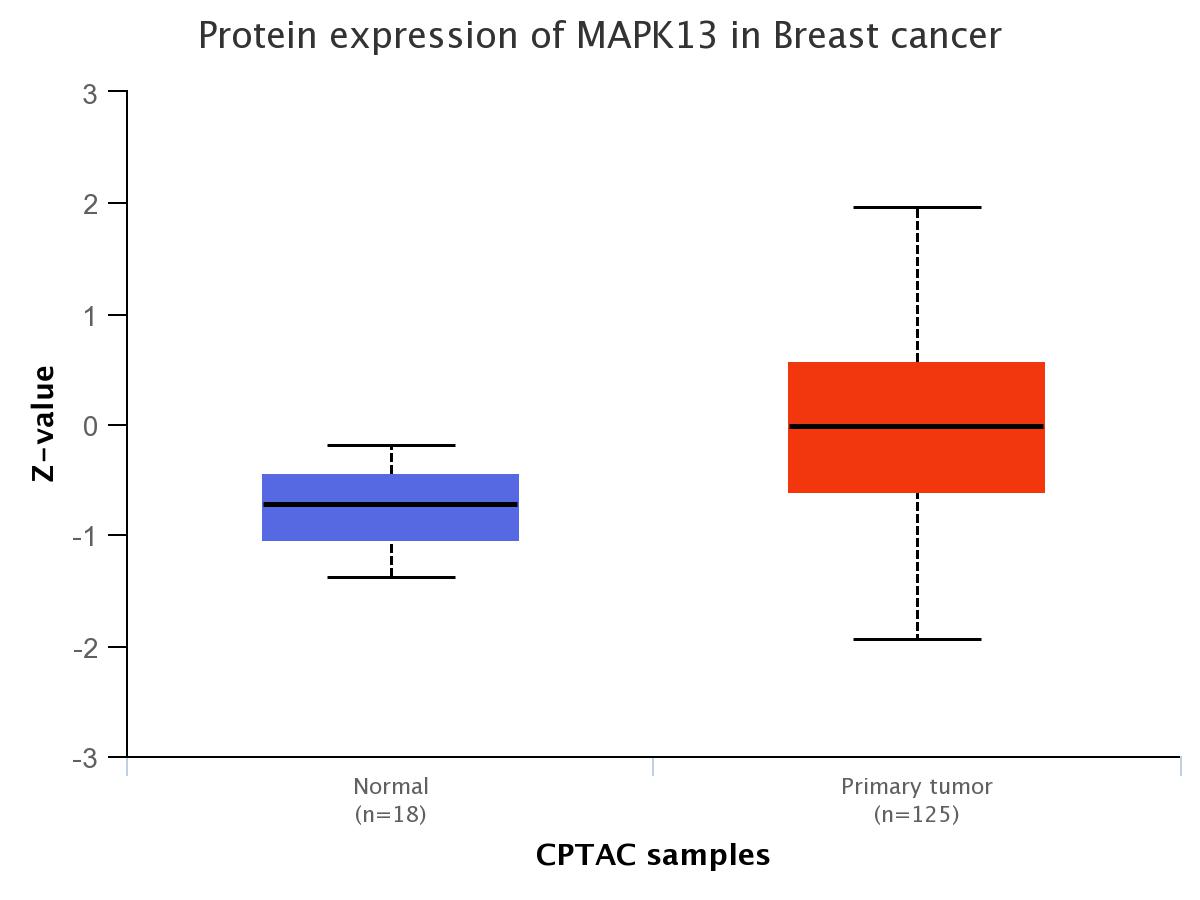


p = 1.0 x 10^-6^

B.


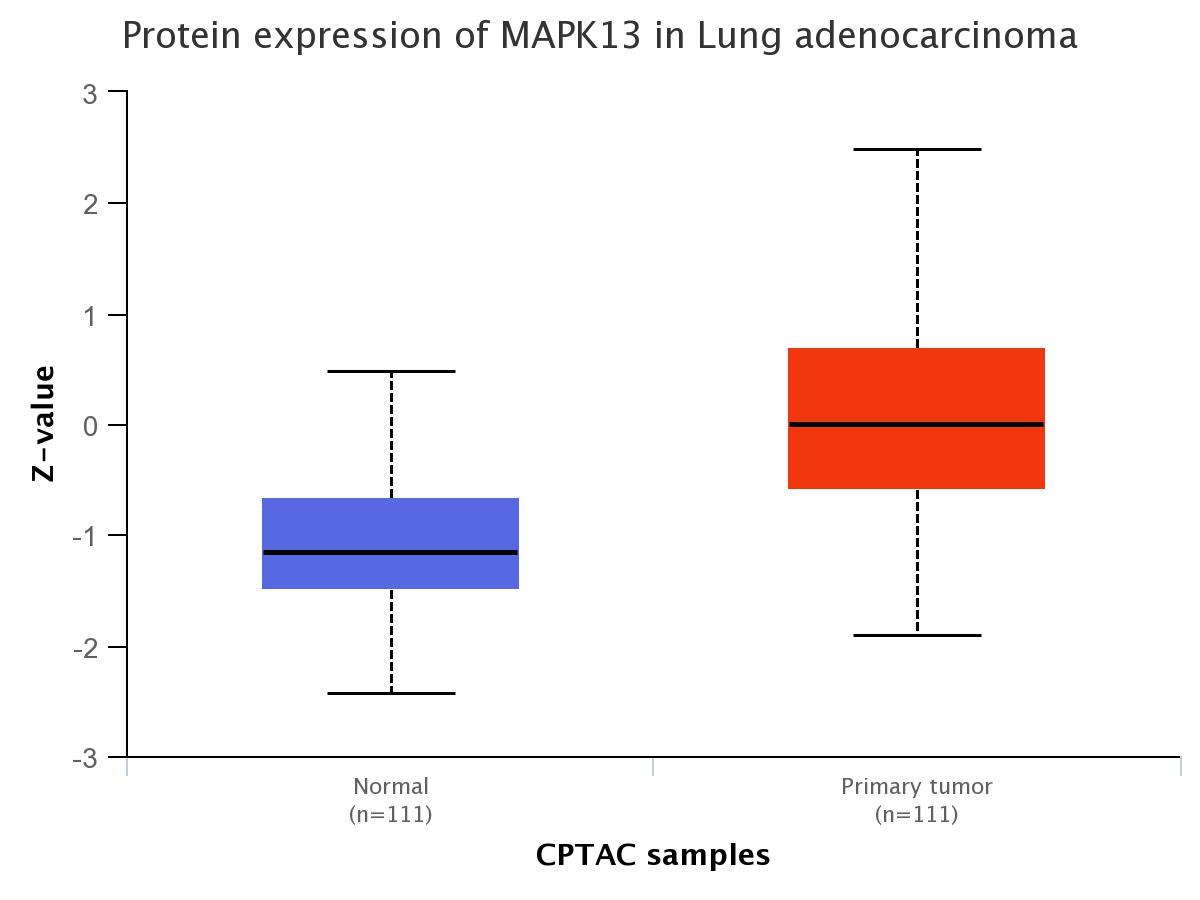


p = 3.3 x 10^-19^

C.

C.


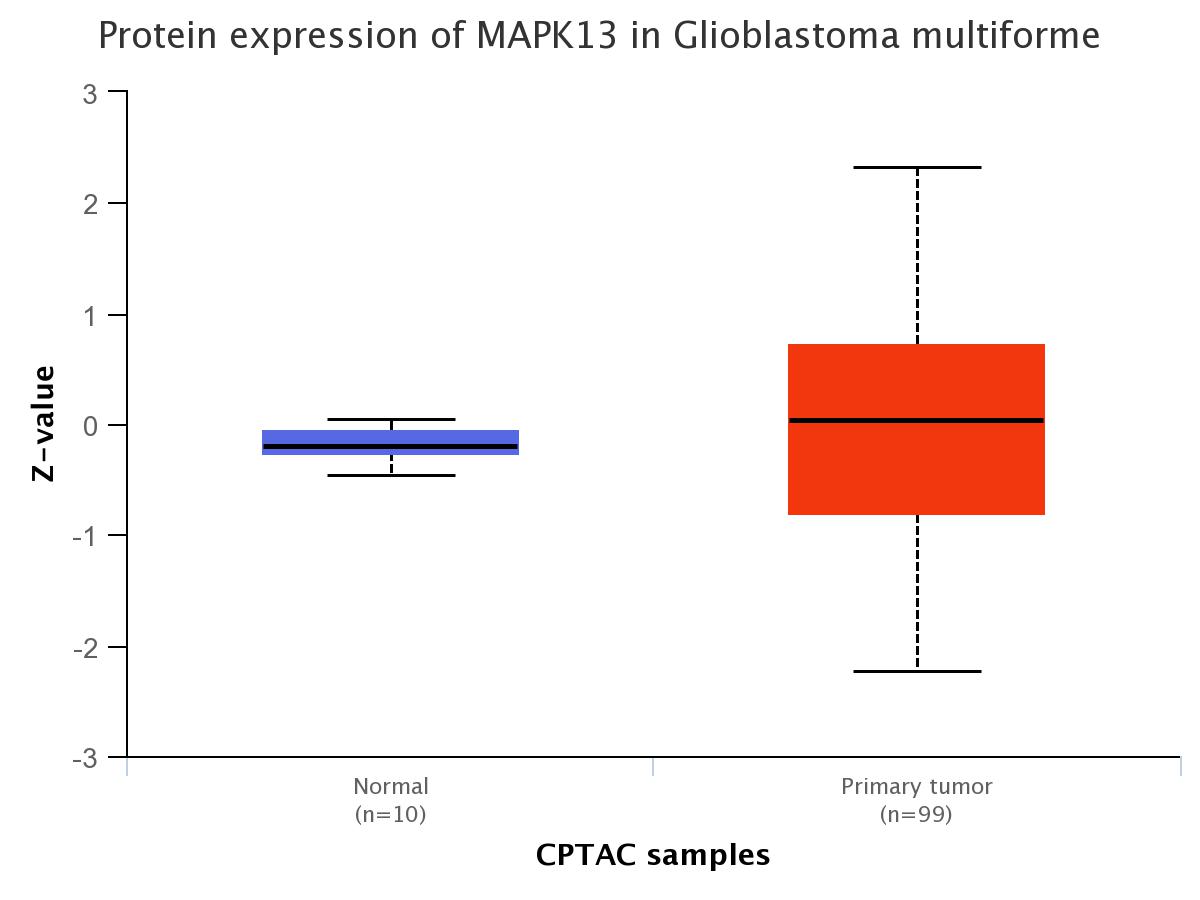


p = 0.7

**Supplemental Figure 3:** Total protein expression levels of MAPK13 in **A.** breast cancer, **B.** lung adenocarcinoma, and **C.** glioblastoma multiforme. CPTAC data was derived from UALCAN.

A.


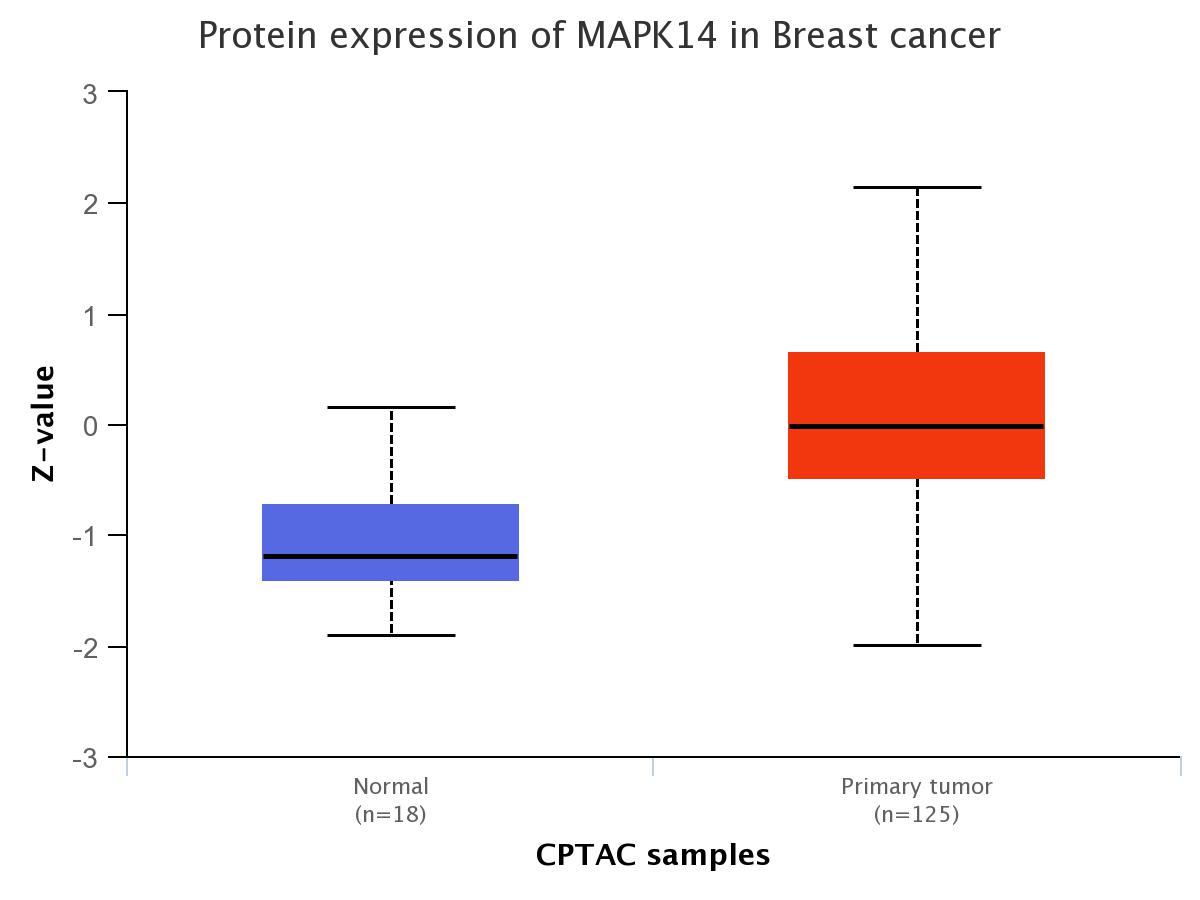


p = 4.9 x 10^-8^

B.


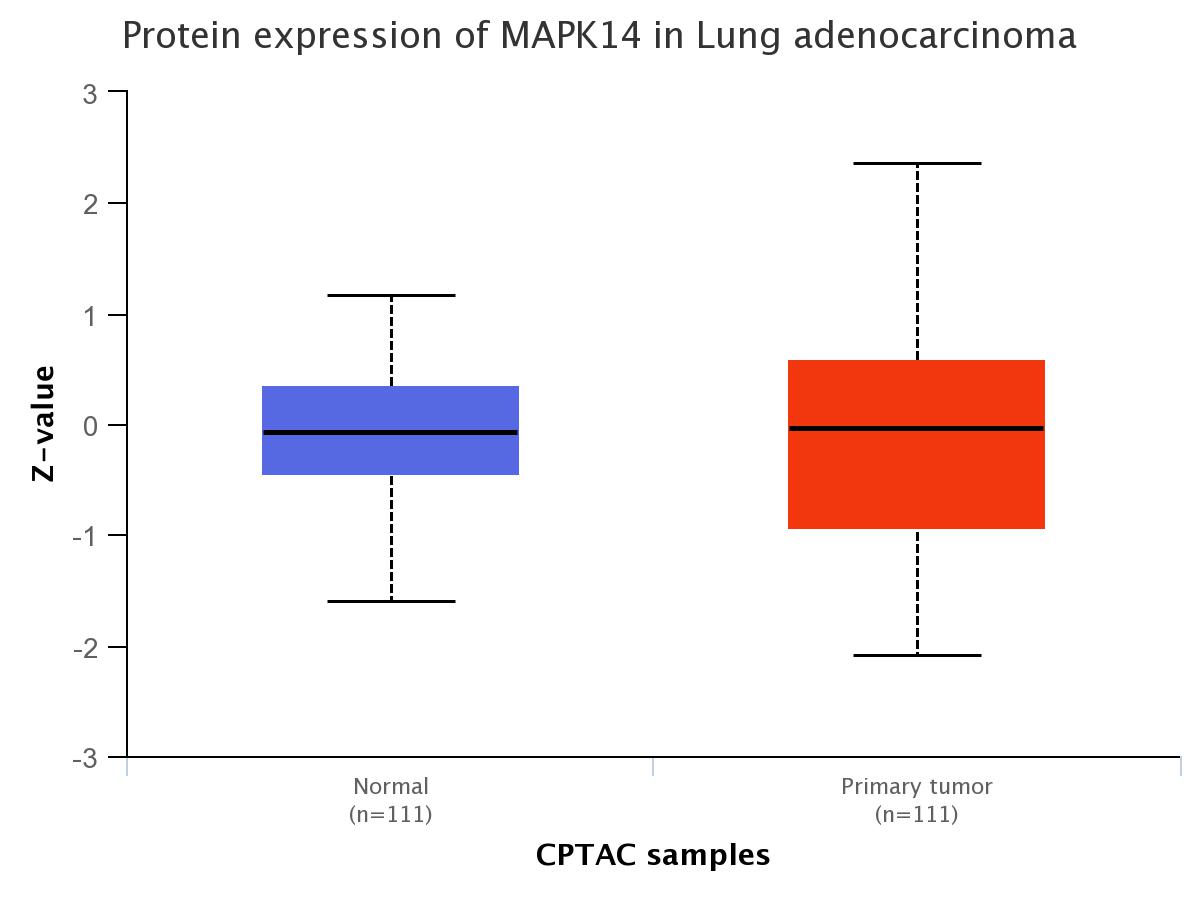


p = 0.8

C.

C.


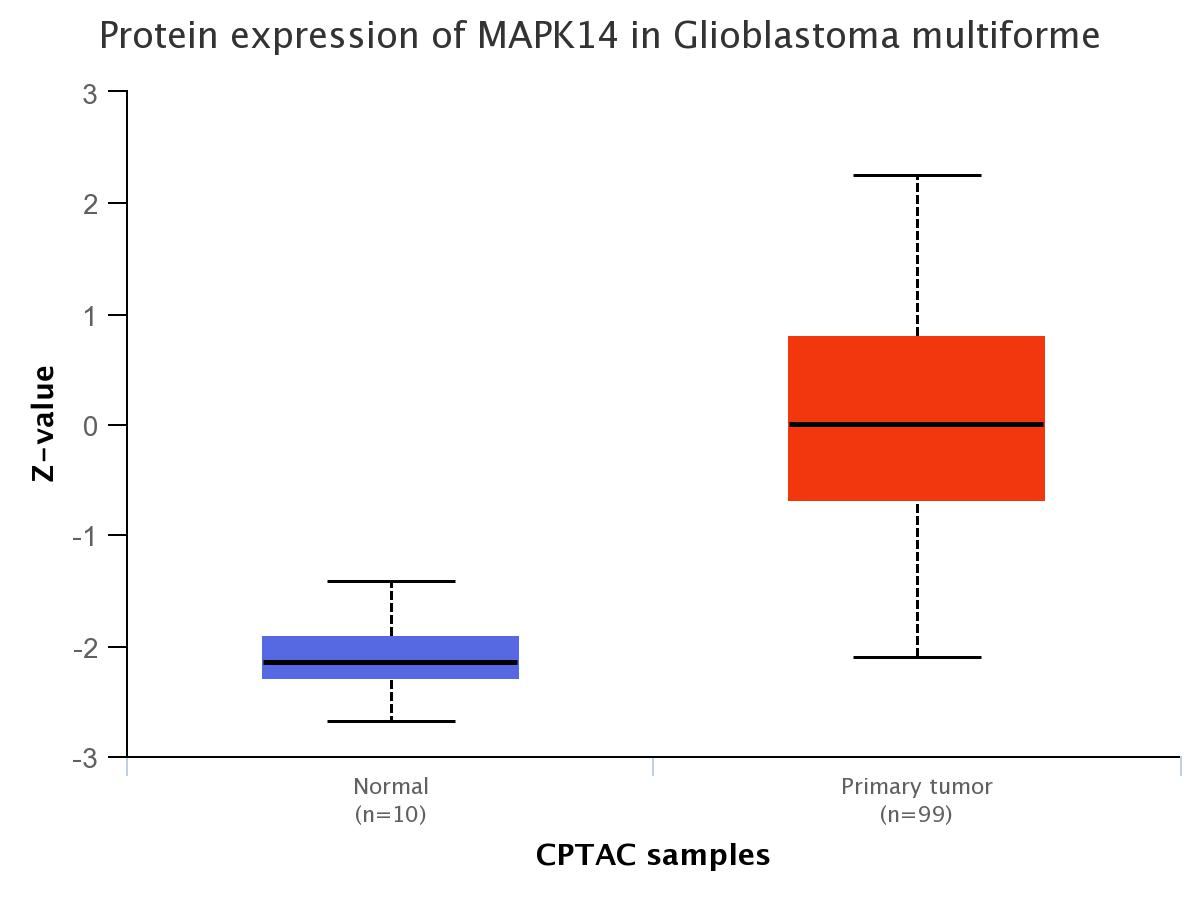


p = 6.2 x 10^-10^

**Supplemental Figure 4:** Total protein expression levels of MAPK14 in **A.** breast cancer, **B.** lung adenocarcinoma, and **C.** glioblastoma multiforme. CPTAC data was derived from UALCAN.

A.


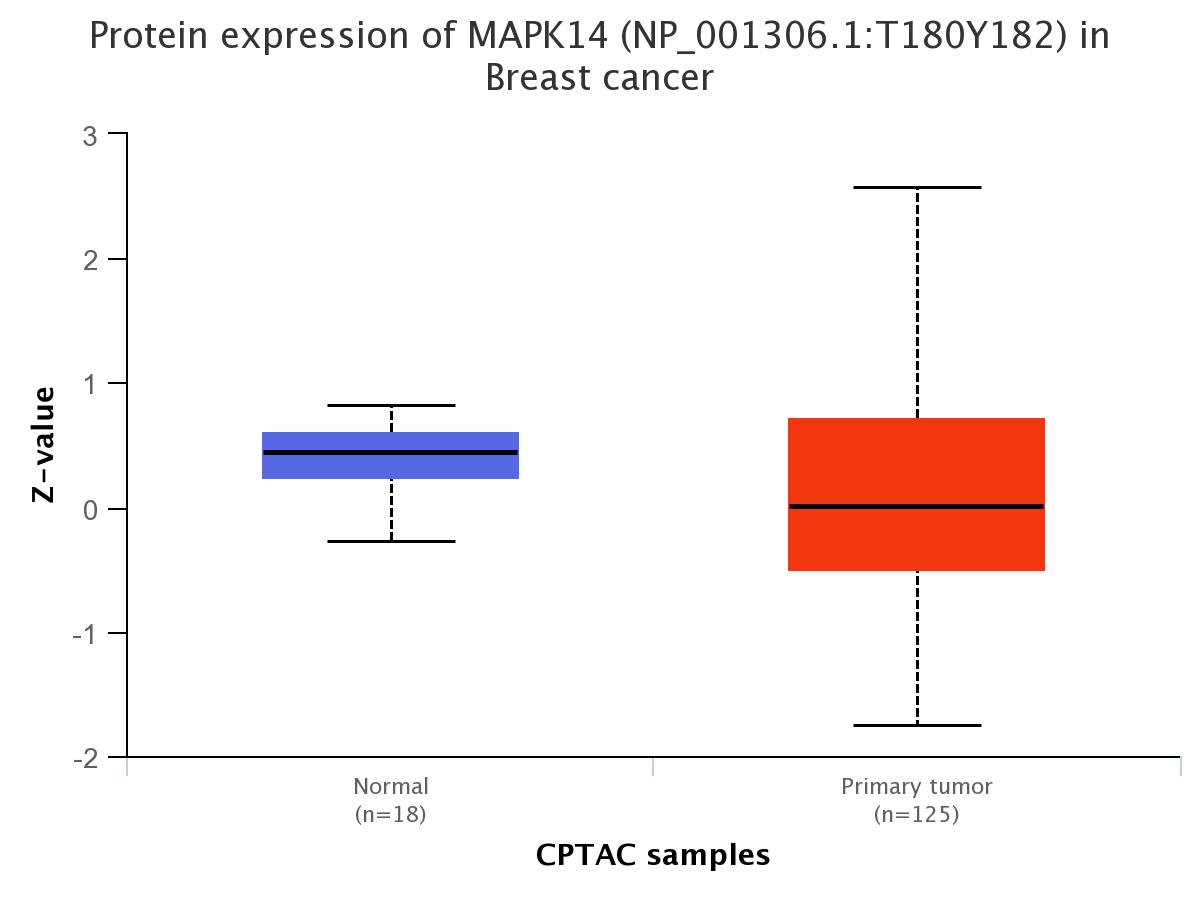


p = 1.1 x 10^-2^

B.


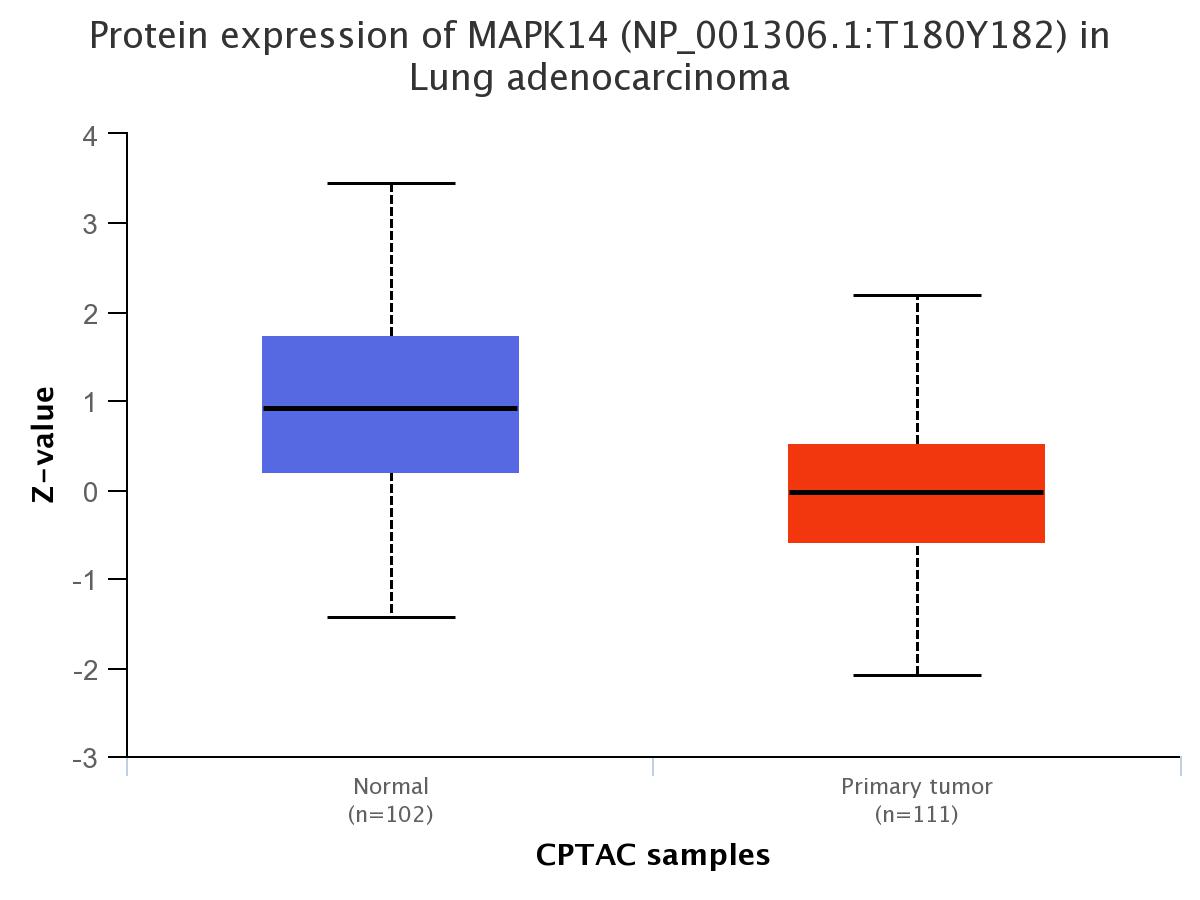


p = 5.5 x 10^-6^

C.


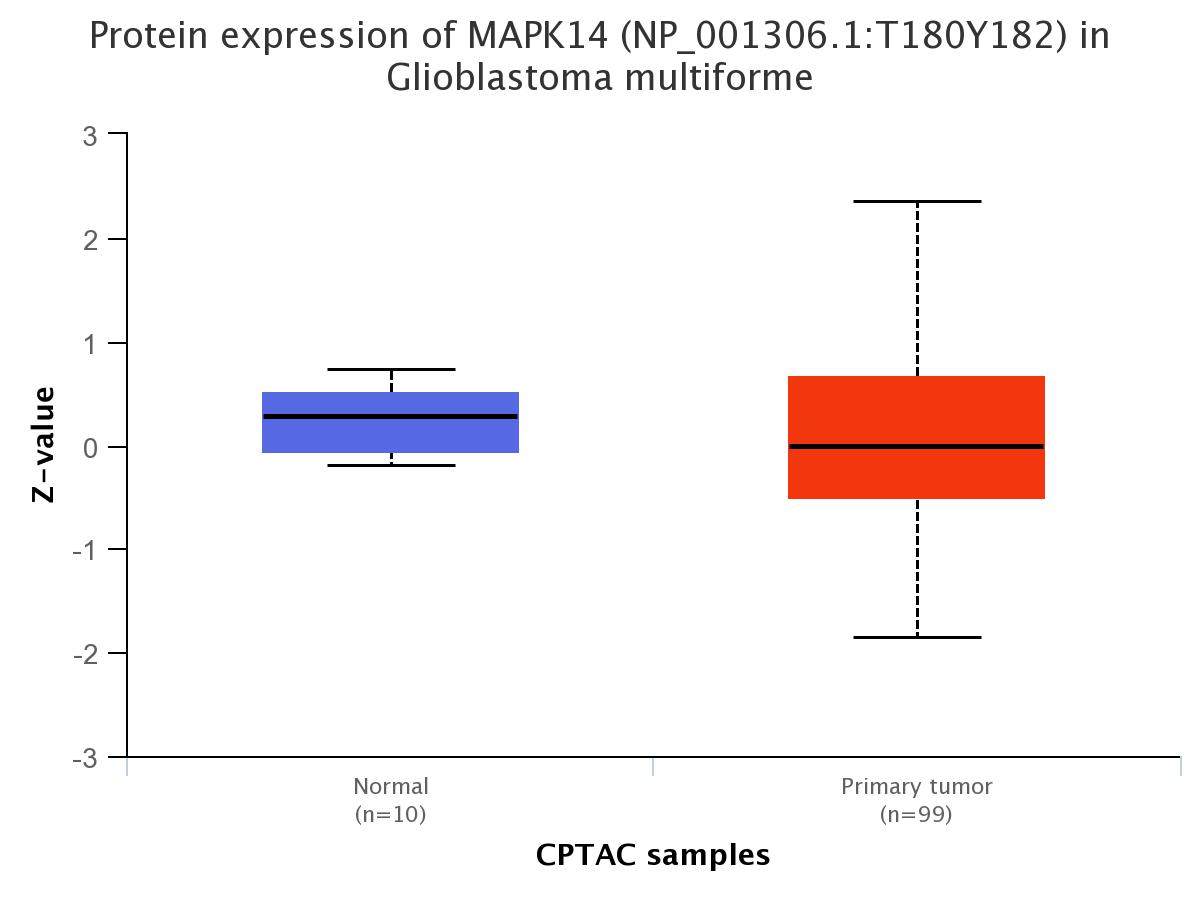


p = 0.1

**Supplemental Figure 5:** Phosphoprotein expression levels of MAPK14 in **A.** breast cancer, **B.** lung adenocarcinoma, and **C.** glioblastoma multiforme. CPTAC data was derived from UALCAN.

A.

B.

**Supplemental Figure 6:** Kaplan-Meier survival curves for breast cancer with **A.** MAPK14 total protein expression and **B.** MAPK14 phosphoprotein expression. Curves were generated using KM Plotter.
